# Synthesis and Photophysical Property Studies of the 2,6,8-Triaryl-4-(phenylethynyl)quinazolines 

**DOI:** 10.3390/molecules19010795

**Published:** 2014-01-10

**Authors:** Malose Jack Mphahlele, Hugues Kadem Paumo, Ahmed M. El-Nahas, Morad M. El-Hendawy

**Affiliations:** 1 Department of Chemistry, College of Science, Engineering and Technology, University of South Africa, P.O. Box 392, Pretoria 0003, South Africa; E-Mail: tkademph@unisa.ac.za; 2 Chemistry Department, Faculty of Science, El-Menoufia University, Shebin El-Kom 32512, Egypt; E-Mail: amelnahas@hotmail.com; 3 Chemistry Department, Faculty of Science, Kafrelsheikh University, Kafrelsheikh 33516, Egypt; E-Mail: Morad.elhendawy@yahoo.com; 4 Higher Institute of Engineering and Technology, Kafrelsheikh 33516, Egypt

**Keywords:** 2-aryl-6,8-dibromoquinazolin-4(3*H*)-ones, 2-aryl-6,8-dibromo-4-chloroquinazolines, Sonogashira cross-coupling, 2-aryl-6,8-dibromo-4-(alkynyl)quinazolines, Suzuki cross-coupling, 2,6,8-triaryl-4-(phenylethynyl)quinazolines, photophysical properties

## Abstract

The 2-aryl-6,8-dibromo-4-chloroquinazolines derived from the 2-aryl-6,8-dibromoquinazolin-4(3*H*)-ones were subjected to the Sonogashira cross-coupling with terminal acetylenes at room temperature to afford novel 2-aryl-6,8-dibromo-4-(alkynyl)quinazoline derivatives. Further transformation of the 2-aryl-6,8-dibromo-4-(phenylethynyl)quinazolines via Suzuki-Miyaura cross-coupling with arylboronic acids occurred without selectivity to afford the corresponding 2,6,8-triaryl-4-(phenylethynyl)quinazolines. The absorption and emission properties of these polysubstituted quinazolines were also determined.

## 1. Introduction

Halogenated quinazolines constitute important substrates for structural elaboration via metal-catalyzed carbon–carbon bond formation to afford novel polysubstituted quinazoline derivatives. It has been established that the order of reactivity of carbon-halogen bonds, C-I > C-Br >> C-Cl, in transition metal-mediated cross-coupling of aryl/heteroaryl halides allows selective coupling with iodides or bromides in the presence of chlorides [1,2]. Although the bond dissociation energy (BDE) of the C-Cl bond at the 4-position (84.8 kcal/mol at B3LYP) of 6-bromo-2,4-dichloroquinazoline is larger than that of the weaker C-Br bond (83 kcal/mol at B3LYP) [3], the selectivity of Pd-catalyzed cross-coupling favours C-4 substitution due to α-nitrogen effect [4,5]. For cross-coupling reactions employing 2,4-dichloroquinazoline, for example, exclusive selectivity for the most electrophilic C-4 position is favoured [3,4,6]. Likewise, regioselective Pd-catalyzed Suzuki-Miyaura cross-coupling reaction of 2,4,7-trichloroquinazoline with aryl- and heteroarylboronic acids favours coupling at C-4 position albeit in low yield due to competitive hydrolysis at this site [7]. Attempts to achieve monosubstitution via the Stille cross-coupling with 6-bromo-2,4-dichloroquinazoline, on the other hand, resulted in mixtures of the C-4 (major) and C-6 (minor) cross-coupled products [4]. However, Sonogashira cross-coupling of 6-bromo-2,4-dichloroquinazoline with stoichiometric amount of terminal alkynes led to exclusive replacement of the 4-chloro atom [5]. During our research on the development of novel polysubstituted heterocycles [8,9], we became interested in the synthesis of polysubstituted quinazolines in which the electron-deficient quinazoline framework is linked to the 4-phenyl ring via π-conjugated spacer and to the 6- and 8-aryl rings directly to comprise donor-π-acceptor systems. We envisioned that the 2-aryl-6,8-dibromo-4-chloroquinazolines represent suitable candidates for sequential Pd-catalyzed Sonogashira and Suzuki cross-coupling to afford the requisite polysubstituted quinazolines with potential photophysical properties.

## 2. Results and Discussion

### 2.1. Synthesis of the 2-Aryl-6,8-dibromoquinazolin-4(3H)-Ones

**Scheme 1:**
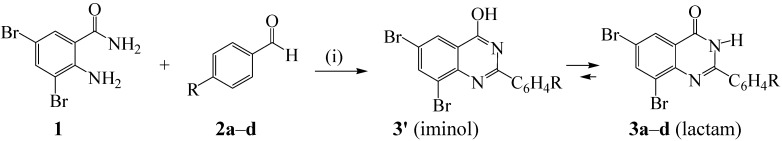
(i) Iodine-promoted cyclocondensation.

The first task was to synthesize the 2-aryl-6,8-dibromoquinazolin-4(3*H*)-ones to serve as substrates for the requisite 2-aryl-6,8-dibromo-4-chloroquinazolines. The potentially tautomeric quinazolin-4(3*H*)-one moiety itself is readily accessible via dehydrogenation of the corresponding 2,3-dihydroquinazolin-4(1*H*)-one precursors using oxidants such as KMnO_4_ [10], CuCl_2_ [11], DDQ [12] and MnO_2_ [13] in stoichiometric or large access. The 2-substituted quinazolin-4(3*H*)-ones have also been synthesized directly from anthranilamide and aldehydes using NaHSO_3_ [14], DDQ [15], CuCl_2_ (3 equiv.) [16], FeCl_3_.6H_2_O [17] or I_2_ [18]. In this investigation, we exploited the combined electrophilic (cyclocondensation) and oxidative (dehydrogenation) properties of iodine on 3,5-dibromobenzamide **1** and benzaldehyde derivatives **2a**–**d** in ethanol under reflux for 7 h to afford products **3a**–**d** in a single-pot operation (Scheme 1). A series of the analogous 2,3-disubstituted 6,8-dibromoquinazolin-4(3*H*)-ones have been prepared before via the reaction of 6,8-dibromo-2-methyl-1-benzoxazin-4(3*H*)-one with nitrogen nucleophiles such as hydrazine hydrate, sulpha drugs and 4-aminoacetophenone [19]. Likewise, the 6-fluoro-8-iodo/bromo-2-methyl-1-benzoxazin-4(3*H*)-ones reacted with aqueous ammonia under reflux to yield the 6-fluoro-8-(iodo/bromo)-2-methylquinazolin-4(3*H*)-ones [20].

*Reagents and conditions*: (i) I_2_ (2 equiv.), ethanol, heat, 7 h.

**Table:** 

	R	%Yield
**3a**	4-H	80
**3b**	4-F	89
**3c**	4-Cl	94
**3d**	4-OMe	91

### 2.2. Oxidative Aromatization of 2-Aryl-6,8-dibromoquinazolin-4(3H)-Ones

**Scheme 2:**
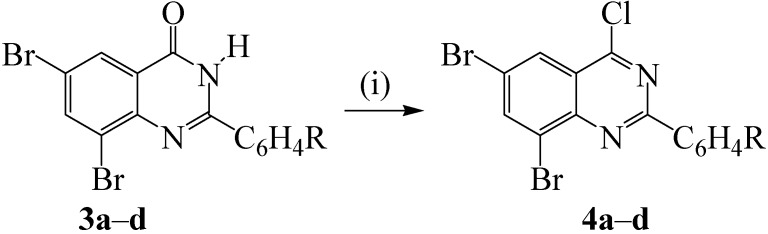
Oxidative aromatization of **3a**–**d** to afford the 4-chloroquinazolines.

The oxidative aromatization of quinazolin-4(3*H*)-one moiety into 4-chloroquinazoline derivatives is often effected by refluxing the NH-4-oxo compound in an excess of POCl_3_ [21] or POCl_3_-PCl_5_ mixture [22]. Oxidative aromatization of compounds **3a**–**d** with POCl_3_, POCl_3_-amine or POCl_3_-DMF mixture under reflux led to incomplete conversion (tlc monitoring) to the requisite 4-chloroquinazolines. The 4-chloroquinazolines **4a**–**d** were prepared in high yields and purity using thionyl chloride in the presence of DMF under reflux for 2 h (Scheme 2).

*Reagents and conditions*: (i) DMF, SOCl_2_, reflux, 2 h.

**Table:** 

	R	% Yield 4
**4a**	4-H	77
**4b**	4-F	81
**4c**	4-Cl	77
**4d**	4-OMe	91

With the halogenated quinazolines **4a**–**d** in hand, we next focused our attention on their reactivity in Sonogashira cross-coupling with terminal alkynes as models for C-C bond formation.

### 2.3. Sonogashira Cross-Coupling of the 2-Aryl-6,8-dibromo-4-chloroquinazolines

**Scheme 3:**
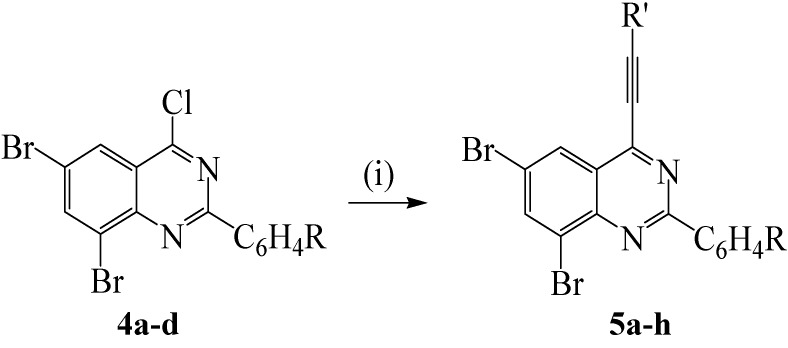
Sonogashira cross-coupling of **4a**–**d** with terminal alkynes.

Sonogashira cross-coupling of **4a** with phenylacetylene (1.5 equiv.) in the presence of tetrakis(triphenylphosphine)palladium(0), CuI and Cs_2_CO_3_ in THF at room temperature for 24 h afforded product **5a**, exclusively. The reaction conditions were extended to other substrates using phenylacetylene, 2-ethynylpyridine and 3-butyn-2-ol to afford products **5b**–**h** (Scheme 3). The analogous 2-substituted quinazolines bearing alkynyl substituent on the C-4 or C-6 position exhibit excellent EGFR or Aurora A kinase inhibition activity [23].

*Reagents and conditions*: (i) R'C≡CH, Pd(PPh_3_)_4_, CuI, Cs_2_CO_3_, THF, rt., 24 h.

**Table:** 

	R	R'	% Yield 5
**5a**	4-H	-C_6_H_5_	60
**5b**	4-F	-C_6_H_5_	72
**5c**	4-Cl	-C_6_H_5_	69
**5d**	4-OMe	-C_6_H_5_	72
**5e**	4-H	2-pyridyl-	65
**5f**	4-F	2-pyridyl-	67
**5g**	4-OMe	2-pyridyl-	53
**5h**	4-H	-CH(OH)CH_3_	56

The presence of the two bromine atoms in compounds **5** makes them suitable candidates for further transformation through transition metal-catalyzed cross-coupling or metal exchange reactions to enable adequate diversity on the heterocycle. This prompted us to explore the reactivity of compounds **5** in palladium catalyzed Suzuki-Miyaura cross-coupling with arylboronic acids as models for C*sp*^2^–C*sp*^2^ bond formation.

### 2.4. Suzuki-Miyaura Cross-Coupling of the 2-Aryl-6,8-dibromo-4-(phenylethynyl)quinazolines

**Scheme 4:**
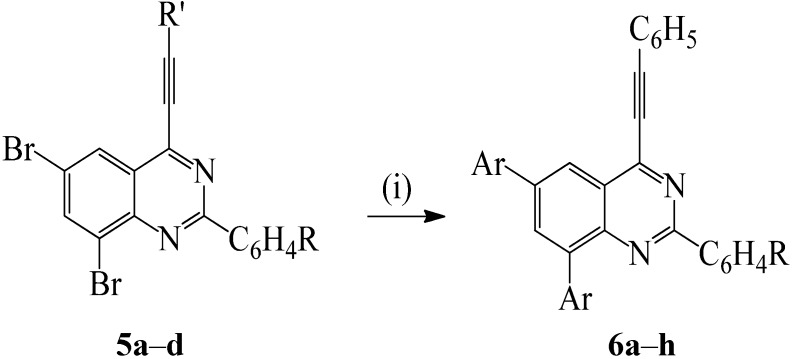
Suzuki-Miyaura cross-coupling of **5a**–**d** with arylvinylboronic acid.

We first subjected compounds **5a**–**d** to 1–1.5 equiv. of the arylboronic acid using PdCl_2_(PPh_3_)_2_/PCy_3_ as catalyst complex, K_2_CO_3_ as a base in dioxane (aq) under reflux. We isolated after 4 h the dicoupled product in low to moderate yields (30–50%) along with the starting material without any traces of the mono cross-coupled derivative. This observation was found to compare with previous literature results for the Suzuki-Miyaura cross-coupling reactions of the analogous 2-arylquinolines bearing two bromine atoms on the fused benzo ring [9] and 3,6,8-tribromoquinoline which occur without selectivity [24]. Computed bond dissociation energies at B3LYP and G3B3 levels reveal that all of the positions on the fused benzo ring of various heterocycles bearing identical halogen atoms have comparable C–X bond dissociation energies [3]. This presumably accounts for the observed lack of selectivity. In analogy with the literature precedents on the analogous di/tribromoquinolines, we opted for the use of an excess arylboronic acid (2.5 equiv.) on compounds **5a**–**d** and we isolated the corresponding tetrasubstituted quinazolines **6a**–**h** in more than 50% yields (Scheme 4).

*Reagents and conditions*: (i) ArB(OH)_2_, PdCl_2_(PCy_3_)_2_, K_2_CO_3_, dioxane (aq), reflux, 4 h.

**Table:** 

	R	Ar	% Yield 6
**6a**	4-H	-C_6_H_5_	61
**6b**	4-F	-C_6_H_5_	69
**6c**	4-Cl	-C_6_H_5_	88
**6d**	4-OMe	-C_6_H_5_	68
**6e**	4-H	4-FC_6_H_4_-	64
**6f**	4-F	4-FC_6_H_4_-	76
**6g**	4-Cl	4-FC_6_H_4_-	77
**6h**	4-OMe	4-FC_6_H_4_-	58
**6i**	4-H	4-MeOC_6_H_4_-	70
**6j**	4-F	4-MeOC_6_H_4_-	62
**6k**	4-Cl	4-MeOC_6_H_4_-	76
**6l**	4-OMe	4-MeOC_6_H_4_-	52

The molecular backbone of compounds **6a**–**l** comprises of the electron-deficient quinazoline framework as an electron-acceptor linked to the 4-phenyl ring via π-conjugated spacer and to the 6- and 8-aryl rings directly to comprise donor-π-acceptor systems.

### 2.5. Photophysical Property Studies of Compounds **6**

To understand the influence of substituents on intramolecular charge transfer (ICT), absorption and emission spectra were measured in solution for compounds **6a**–**l**. Electronic properties of compounds **6a**–**l** were studied by UV/Vis and fluorescence spectroscopy in conjunction with quantum chemical calculations to establish the effect of substituents on the absorption and emission properties of these polysubstituted quinazoline derivatives.

#### 2.5.1. UV-Vis Absorption Properties of the 2,6,8-Triaryl-4-(phenylethynyl)quinazolines **6a**–**l**

**Figure 1:**
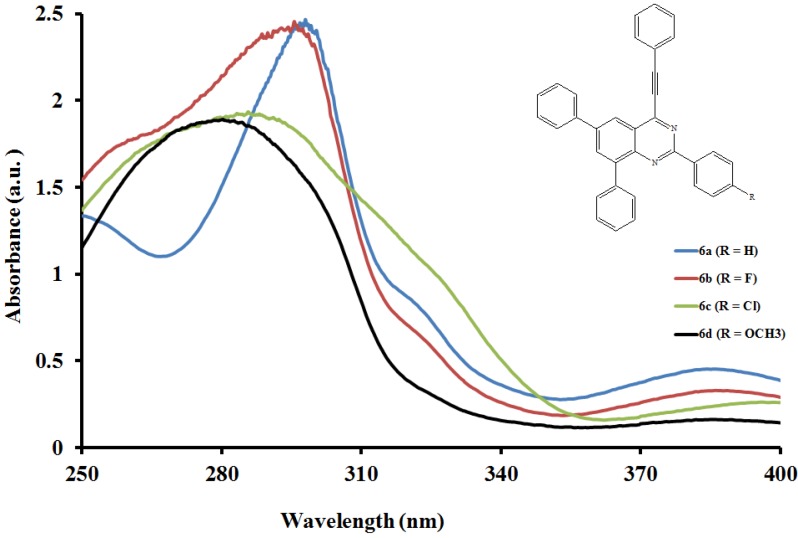
UV-Vis spectra of **6a**–**d** in CHCl_3_ (0.022 mmol/L).

**Figure 2:**
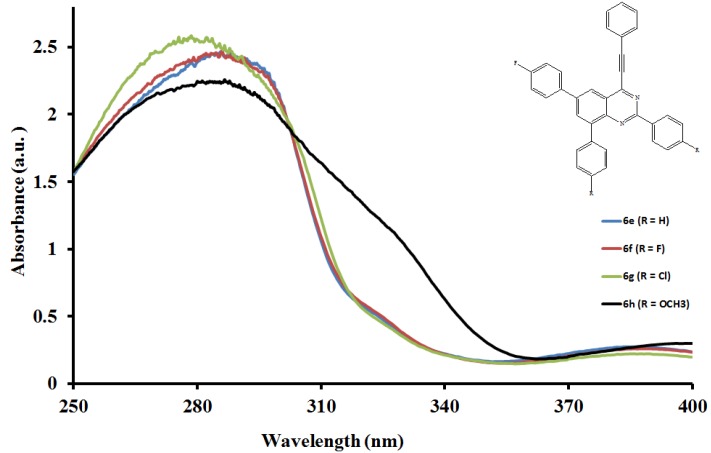
UV-Vis spectra of **6e**–**h** in CHCl_3_ (0.022 mmol/L).

**Figure 3:**
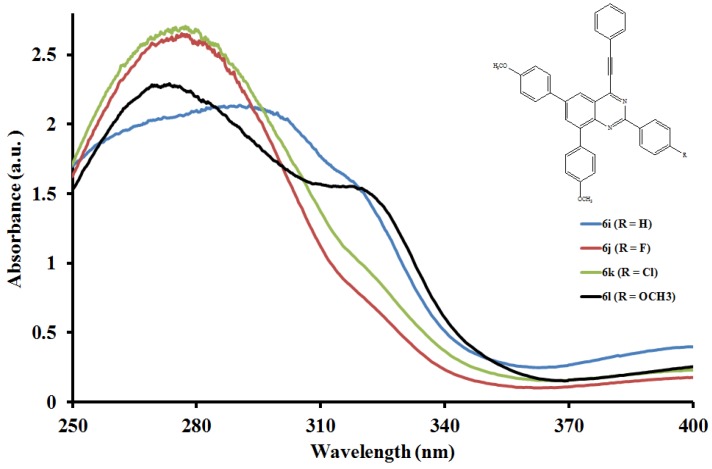
UV-Vis spectra of **6i**–**l** in CHCl_3_ (0.022 mmol/L).

The electronic absorption spectra of compounds **6a**–**l** (Figure 1, Figure 2 and Figure 3) were acquired in CHCl_3_ and are characterized by intense broad bands in the ultraviolet region λ 270–295 nm. These bands are attributed to the π-π* transition and the intramolecular donor-acceptor charge transfer absorption, respectively [25]. Both the absorption maxima and wavelength within each series are influenced by the variation of substituents on the *para* position of the aryl groups on the fused benzo ring and the 2-aryl substituents. In the case of the 2-phenyl derivatives **6a**, **6e** and **6i**, intensity of the absorption bands decreases with increasing conjugative effect of the substituent on the 2-aryl ring, **6a** > **6e** > **6i**, and is accompanied by the reverse trend in peak broadening (**6a** < **6e** < **6i**). Moreover, the absorption wavelengths for **6e** and **6i** bearing the moderately and strongly donating 4-fluorophenyl- and 4-methoxyphenyl substituents are blue shifted relative to **6a**. Increased intensity of the absorption maxima is observed in the spectra of all the 2-(4-fluorophenyl) substituted derivatives **6b**, **6f** and **6j**. The trend in molar extinction coefficients, **6j** > **6f** > **6b**, reflects the electron donating effect of the 6- and 8-aryl rings. The presence of a strong electron withdrawing 2-(4-chlorophenyl) group in compounds **6g** and **6k** causes the moderately resonance donating 4-fluorophenyl and strongly donating 4-methoxyphenyl groups to increase the π–π* transition resulting in increased absorption intensities for these compounds. Reduced intensity of the absorption maxima due to reduced π–π* transition accompanied by increased broadening are observed for **6c** bearing the 6- and 8-phenyl groups. A combination of the 2-(4-methoxyphenyl) substituent with phenyl groups in **6d** or with the 4-methoxyphenyl groups at the 6- and 8-positions in compounds **6h** and **6l** resulted in reduced intensity of the absorption maxima and increased broadening. Increased peak broadening and reduced intensity indicate that the strong electron donating methoxy groups interfere with the conjugation of the π electrons presumably restricting the transition from bonding orbital to antibonding orbital. The additional low intensity band observed for compound **6l** at μ *ca.* 320 nm is probably the consequence of poor through-space charge transfer by the strongly electron donating 4-methoxyphenyl groups at the 6- and 8-positions.

#### 2.5.2. Emission properties of the 2,6,8-triaryl-4-(phenylethynyl)quinazolines **6a**–**l**

**Figure 4:**
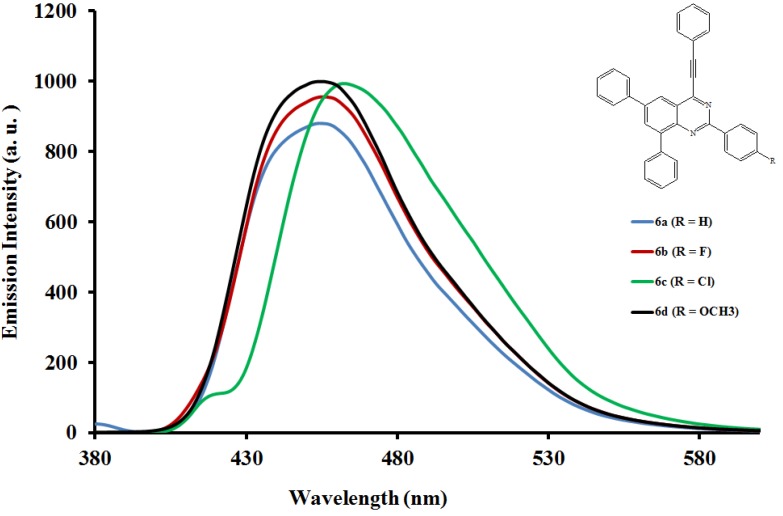
The emission spectra of compounds **6a**–**d** (λex = 380 nm) in CHCl_3_ (0.022 mmol/L) at rt.

**Figure 5:**
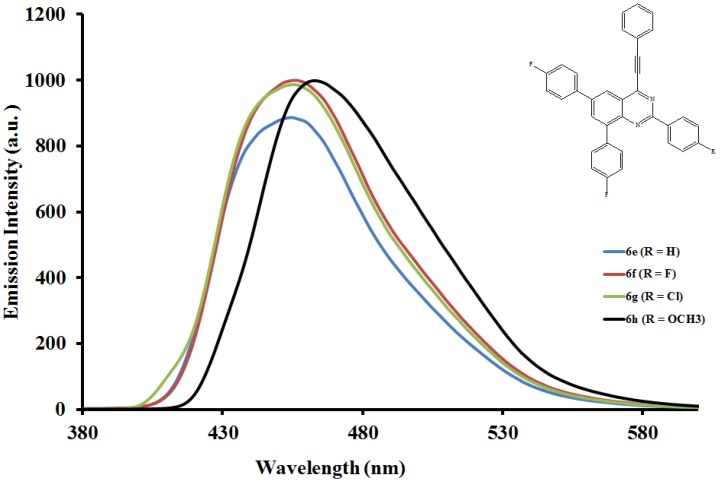
The emission spectra of compounds **6e**–**h** (λex = 380 nm) in CHCl_3_ (0.022 mmol/L) at rt.

**Figure 6:**
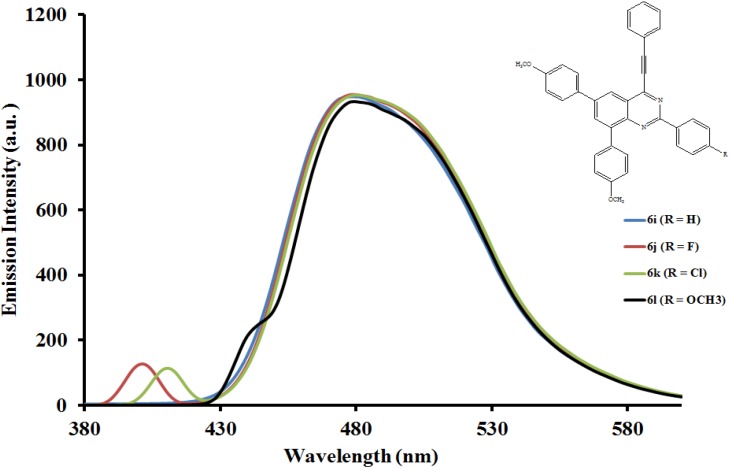
The emission spectra of compounds **6i**–**l** (λex = 380 nm) in CHCl_3_ (0.022 mmol/L) at rt.

**Figure 7:**
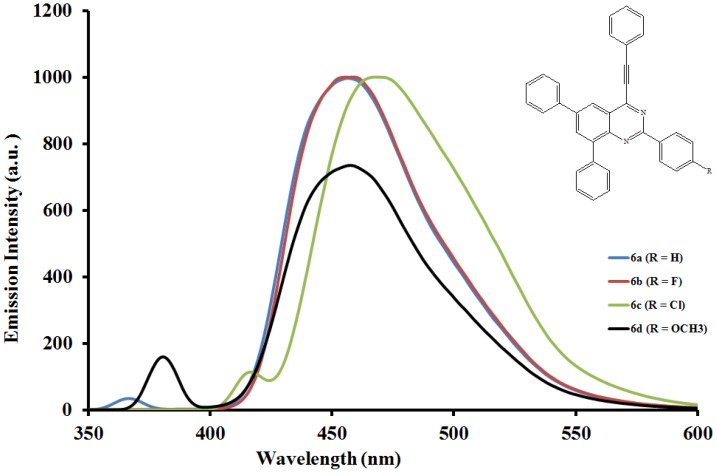
The emission spectra of compounds **6a**–**d** (λex = 400 nm) in DMF (0.022 mmol/L) at rt.

**Figure 8:**
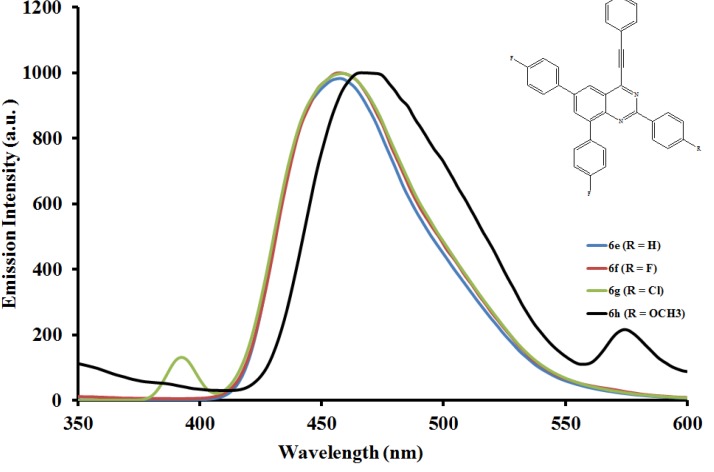
The emission spectra of compounds **6e**–**h** (λex = 400 nm) in DMF (0.022 mmol/L) at rt.

**Figure 9:**
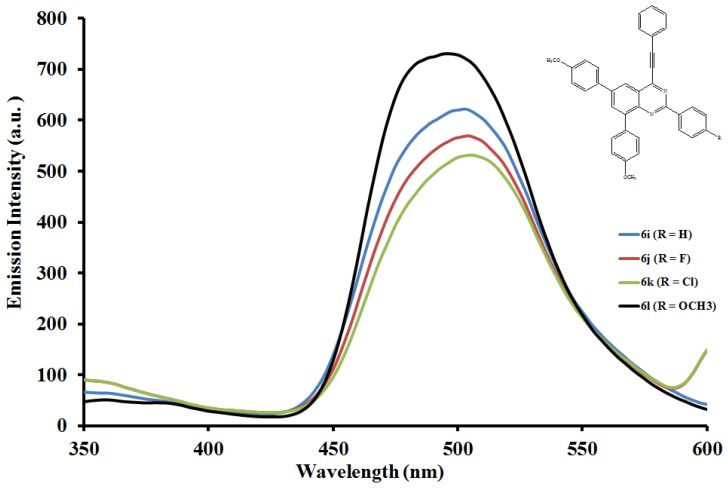
The emission spectra of compounds **6i**–**l** (λex = 400 nm) in DMF (0.022 mmol/L) at rt.

The emission properties of compounds **6a**–**l** have also been studied at room temperature in the moderately polar chloroform (Figure 4, Figure 5 and Figure 6) and strongly polar DMF (Figure 7, Figure 8 and Figure 9) at the excitation wavelengths, λ_ex_ = 380 nm and 400 nm, respectively. Their emission spectra in both solvents are characterized by intense single emission bands attributed to increased π-π* transition resulting from direct π-electron delocalization by the aryl groups and through the conjugate bridge towards the electron-deficient quinazoline ring. Moreover, within each series the emission wavelengths, Stokes shift and the fluorescence quantum yields are influenced by the variation of substituents on either the *para* position of the 2-aryl or the 6- and 8-aryl groups (Table 1). Compounds **6a** and **6e**, for example, exhibit relatively reduced emission intensities in CHCl_3_ than **6i** bearing the 4-methoxyphenyl groups at 6- and 8-positions. Moreover, the following trends in Stokes shift and quantum yields: **6i** > **6e** > **6a** are consistent with the conjugative effects of the 6- and 8-aryl substituents. Likewise, compound **6j** exhibits larger Stokes shift than **6b** and **6f**, but with lower quantum yield.

Since the π,π* state is much more polarizable than the ground state, a change in polarity of the medium has been previously found to cause measurable displacements of the π-π* transition towards the red bands [26]. The emission spectra of compounds **6a**–**l** in DMF are also characterized by intense single emission bands (Figure 7, Figure 8 and Figure 9). For the 4-phenylethynylquinazolines **6a**, **6f** and **6l** bearing similar aryl groups at the 2-, 6- and 8-positions, the presence of strongly electron donating 4-methoxyphenyl groups in compound **6l** leads to reduced emission intensity, which is accompanied by higher emission wavelength. A similar trend in intensity is observed for compounds **6c**, **6g** and **6k** bearing a strong electron withdrawing chloro group on the 2-phenyl ring. However, a combination of the 2-(4-chlorophenyl) and 6- and 8-(4-fluorophenyl) groups leads to decreased emission wavelength. For the 2-(4-methoxyphenyl) derivatives **6d**, **6h** and **6l** the emission intensity seems to be influenced by the electron donating effect of the 6- and 8-(4-ethoxyphenyl) rings. Additional interaction of DMF with the methoxy group of **6d** would reduce the propensity of the 2-(4-methoxyphenyl) substituent for π-electron pair delocalization into the quinazoline ring. Such interaction would probably result in relatively less pronounced ICT and therefore reduced maxima for **6d** (Figure 7). Relatively increased emission maxima observed in the spectra of compounds **6h** (Figure 8) and **6l** (Figure 9) in DMF are presumably due to increased π-electron delocalization into the quinazoline ring by the moderately and strongly donating 4-fluorophenyl and 4-methoxyphenyl groups, respectively. A combination of the 2-(4-methoxyphenyl) group and the 4-fluorophenyl groups at the 6- and 8-positions in **6h**, on the other hand, leads to red shift of the emission maxima (Figure 8). An additional undesired red-shifted emission band of reduced intensity exhibited by **6h** in DMF is presumably due to the re-absorption of light emitted and/ or molecular excited state interaction with a ground state molecule leading to a partial transfer of charge in the molecule [27]. The emission spectra of compounds **6i**–**l** showed pronounced red shifts with increasing solvent polarity and the intensities of their emission maxima in DMF seem to be influenced by the electronic effect of the substituent on the *para* position of the 2-phenyl group: MeO>H>F>Cl (Figure 9). The solvent-dependent emission characteristics may result from the dipolar interaction with DMF thus suggesting the ICT character of the emission state [28].

**Table 1:** The absorption and emission data for compounds **6a**–**l**.

Compounds	λ_max_ (nm) CHCl_3_	(ε) × 10^3^ Mol^−1^cm^−1^	λ_em_ (nm) CHCl_3_	λ_em_ (nm) DMF	^(a)^ Quantum yields (Φ)	Stokes shift CHCl_3_
**6a**	298.0	11.216	454.5	456.5	0.071	156.5
**6b**	295.6	11.163	455.0	459.0	0.078	159.4
**6c**	285.7	8.798	462.5	467.0	0.102	176.8
**6d**	280.3	8.601	455.0	458.0	0.105	174.7
**6e**	285.7	11.216	454.5	457.5	0.071	168.8
**6f**	284.5	11.181	456.0	457.0	0.081	171.5
**6g**	278.5	11.754	455.0	458.5	0.076	176.5
**6h**	286.6	10.271	463.0	466.5	0.088	176.4
**6i**	292.9	9.725	478.5	503.0	0.088	185.6
**6j**	276.4	12.063	479.0	504.5	0.072	202.6
**6k**	276.7	12.268	480.0	505.5	0.070	203.3
**6l**	273.4	10.422	479.5	496.0	0.081	206.1

^(a)^ The relative quantum yields in CHCl_3_ were calculated according to the equation indicated under Experimental section using quinine sulfate as the standard (Φ_q_ = 0.55) in 0.5 M H_2_SO_4_.

#### 2.5.3. Quantum Chemical Calculations

**Figure 10:**
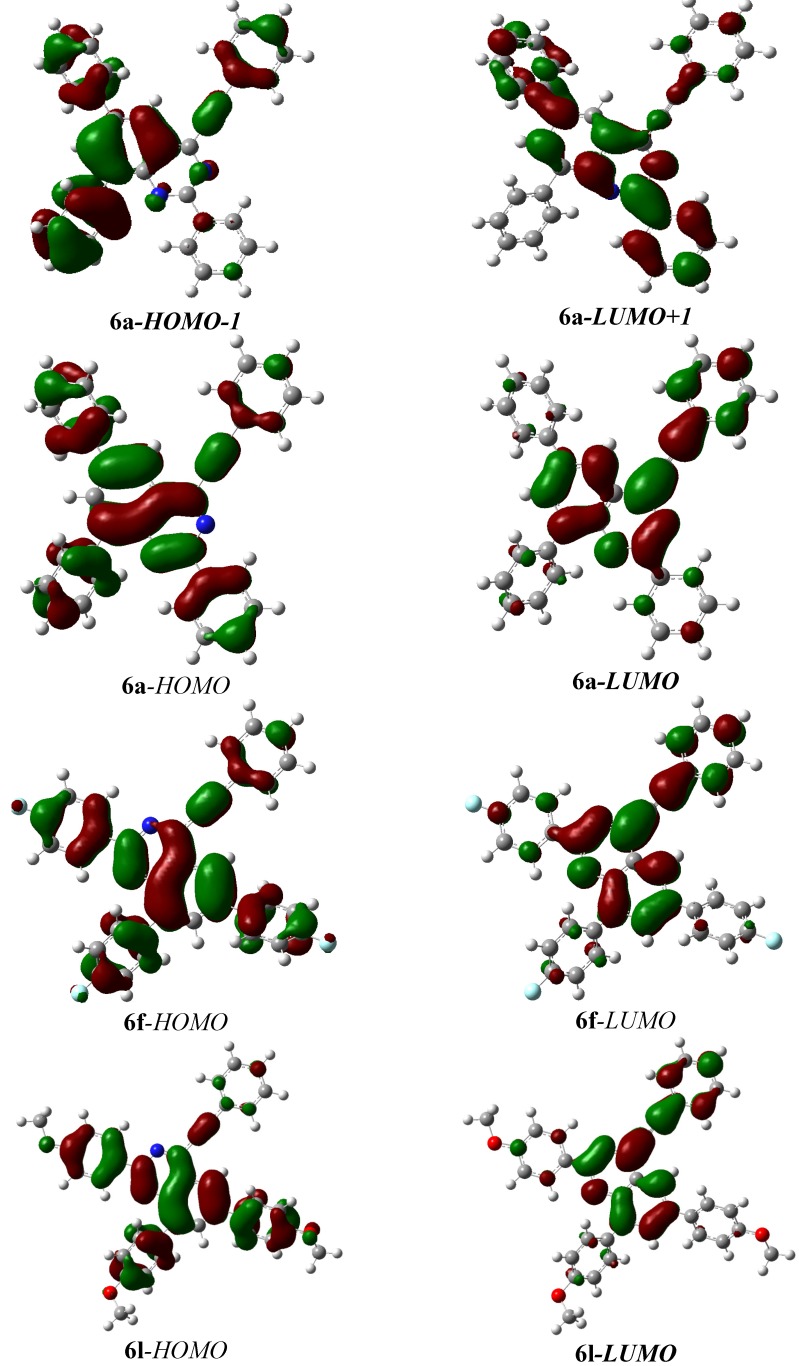
Selected frontier molecular orbitals, HOMO-1, HOMO, LUMO and LUMO+1 of some representative compounds.

To further establish the structural features and molecular orbitals of compounds **6**, we carried out a theoretical approach using density functional theory at the B3LYP/6-31G* level. The geometries were optimized using CAM-B3LYP/6-31G(d,p) [29] as implemented in Gaussian 09 suite [30] to obtain reasonable structures for the subsequent electronic structure computations. Based upon the CAM-B3LYP geometries, single-point ZINDO/S calculations were preformed in chloroform as inexpensive, rapid and relatively accurate computations [31]. Compound **6a** was chosen as a representative model to assign the absorption bands in the electronic spectra. The lowest energy band at 373 nm which represents S_1_ state has moderate oscillator strength of *ca.* 0.5. This band is assigned mainly to the electronic transition between the frontier orbitals, where the HOMO→LUMO transition is the main contribution to the first excited state (S_1_). The HOMO is delocalized over the entire molecule whereas the LUMO shrinks toward the quinazoline core (Figure 10) and these represent π and π* orbitals, respectively. The band located at 328 nm with oscillator strength of 0.3 is based on S_4_ singlet state. This state consists of HOMO→LUMO (55%), HOMO-1→LUMO (15%) and HOMO→LUMO+1 (15%). The HOMO-1 is mainly localized over the 6- and 8-aryl groups, while the LUMO-1 is mainly localized on the quinazoline framework, 2- and 8-aryl moieties. The most intense band at 298 nm, on the other hand, arises from the electronic excitation to S_7_ singlet state, which consists predominantly of HOMO-1→LUMO (47%) and HOMO→LUMO+1 (25%) transitions. Similar results were observed for the other compounds. Figure 10 shows the HOMO and LUMO of **6a**, **6f** and **6k** and no great changes were observed on the electron density distributions.

**Figure 11:**
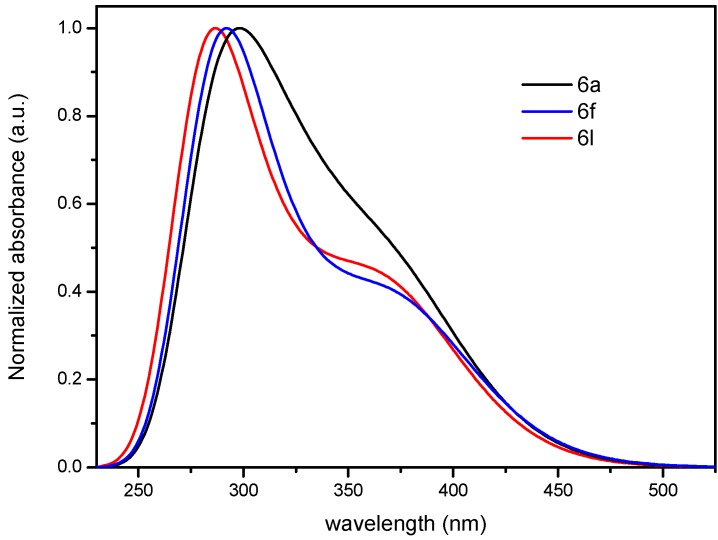
The computed electronic absorption spectra of **6a**, **6f** and **6l.**

The computed spectral profiles of **6a**, **6f** and **6k** were chosen and are presented in Figure 11 in order to reveal the effect of substitution on the *para* position of the 2-, 6- and 8-phenyl groups on the electronic spectra. The presence of electron donating group on the *para* positions of the phenyl ring causes a blue shift (*ca.* 15 nm) in compounds **6a** to **6l**. The calculated spectral data compares favourably with the experimental ones. The 4-methoxy groups in compound **6l**, on the other hand, enhance the intramolecular charge transfer from the aryl groups into the quinazoline core more than the 4-fluoro substituents in **6f** and the parent compound **6a**. This could explain the appearance of CT-band in **6l** compared to **6f**. In the case of **6a**, the CT-band at the longer wavelength is merged with the most intense band presumably due to the relatively poor resonance donation by the phenyl groups that are not able to make charge separation with the acceptor core.

## 3. Experimental

### 3.1. General

Melting points were recorded on a Thermocouple digital melting point apparatus and are uncorrected. IR spectra were recorded as powders using a Bruker VERTEX 70 FT-IR Spectrometer with a diamond ATR (attenuated total reflectance) accessory by using the thin-film method. For column chromatography, Merck kieselgel 60 (0.063–0.200 mm) was used as stationary phase. The UV-vis spectra were recorded on a Cecil CE 9500 (9000 Series) UV-Vis spectrometer while emission spectra were taken using a Perkin Elmer LS 55 fluorescence spectrometer. The quantum efficiencies of fluorescence (Φ_fl_) were obtained with the following equation:

Φ_x_ = Φ_st_*(*F_x_*/*F_st_*)*(A_st_/A_x_)*(*n*_x_^2^/*n*_st_^2^)

*F* denotes the area under the fluorescence band (*F = ^a^I*_fl_(λ), where *I*_fl_(λ) is the fluorescence intensity at each emission wavelength), A denotes the absorbance at the excitation wavelength, and *n* is the refractive index of the solvent [32]. NMR spectra were obtained as DMSO-*d*_6_ or CDCl_3_ solutions using Varian Mercury 300 MHz NMR spectrometer and the chemical shifts are quoted relative to the solvent peaks. Low- and high-resolution mass spectra were recorded at the University of Stellenbosch Mass Spectrometry Unit using Synapt G2 Quadrupole Time-of-flight mass spectrometer.

### 3.2. I_2_–Promoted Cyclocondensation of 3,5-Dibromoanthranilamide and Arylaldehydes

#### Typical Procedure

A stirred mixture of 2-amino-3,5-dibromobenzamide **1** (1 equiv.), benzaldehyde derivative **2** (1.4 equiv.) and iodine (2 equiv.) in ethanol (20 mL per mmol of **1**) was refluxed for 7 h. The mixture was allowed to cool to room temperature and then quenched with an ice-cold saturated sodium thiosulfate solution. The resulting precipitate was filtered on a sintered funnel and then washed with an ice-cold water. The solid product was recrystallized from acetonitrile to afford the corresponding quinazolin-4(3*H*)-one **3**. The following products were prepared in this fashion:

*6,8-Dibromo-2-phenylquinazolin-4(3H)-one* (**3a**). A mixture of **1** (1.00 g, 3.37 mmol), benzaldehyde (**2a**) (0.43 g, 4.06 mmol) and iodine (1.19 g, 6.74 mmol) in ethanol (100 mL) afforded **3a** as a white solid (1.08 g, 80%), m.p. 332–335 °C; ν_max_ (ATR) 698, 742, 1147, 1361, 1486, 1599, 1681, 3174, 3380 cm^−1^; δ_H_ (300 MHz, DMSO-*d*_6_) 7.56–7.64 (m, 3H), 8.21–8.25 (m, 2H), 8.26 (d, *J* = 3.0 Hz, 1H), 8.38 (d, *J* = 3.0 Hz, 1H), 12.93 (s, 1H); *m/z* 379 (100, MH^+^); HRMS (ES): MH^+^, found 378.9087. C_14_H_9_N_2_O^79^Br_2_^+^ requires 378.9082.

*6,8-Dibromo-2-(4-fluorophenyl)quinazolin-4(3H)-one* (**3b**). A mixture of **1** (1.00 g, 3.37 mmol), 4-fluorobenzaldehyde (**2b**) (0.50 g, 4.06 mmol) and iodine (1.90 g, 6.74 mmol) in ethanol (100 mL) afforded **3c** as a white solid (1.20 g, 89%), m.p. > 350 °C; ν_max_ (ATR) 875, 1155, 1230, 1487, 1599, 1686, 3374 cm^−1^; δ_H_ (300 MHz, DMSO-*d*_6_) 7.43 (t, *J* = 8.7 Hz, 2H), 8.19 (d, *J* = 3.0 Hz, 1H), 8.31 (t, *J* = 8.7 Hz, 2H), 8.34 (d, *J* = 3.0 Hz, 1H), 12.94 (s, 1H); *m/z* 397 (100, MH^+^); HRMS (ES): MH^+^, found 396.8975. C_14_H_8_N_2_OF^79^Br_2_^+^ requires 396.8987.

*6,8-Dibromo-2-(4-chlorophenyl)quinazolin-4(3H)-one* (**3c**). A mixture of **1** (1.00 g, 3.37 mmol), 4-chlorobenzaldehyde (**2c**) (0.56 g, 4.06 mmol) and iodine (1.90 g, 6.74 mmol) in ethanol (100 mL) afforded **3c** as a white solid (1.37 g, 94%), m.p. > 350 °C; ν_max_ (ATR) 724, 823, 1408, 1481, 1671, 3138, 3362 cm^−1^; δ_H_ (300 MHz, DMSO-*d*_6_) 7.64 (d, *J* = 9.3 Hz, 2H), 8.19 (d, *J* = 3.0 Hz, 1H), 8.27 (d, *J* = 9.3 Hz, 2H), 8.34 (d, *J =* 3.0 Hz, 1H), 12.98 (s, 1H); *m/z* 413 (100, MH^+^); HRMS (ES): MH^+^, found 412.8683. C_14_H_8_N_2_O^35^Cl^79^Br_2_^+^ requires 412.8692.

*6,8-Dibromo-2-(4-methoxyphenyl)quinazolin-4(3H)-one* (**3d**). A mixture of **1** (1.00 g, 3.37 mmol), 4-methoxybenzaldehyde (**2d**) (0.54 g, 4.06 mmol) and iodine (1.90 g, 6.74 mmol) in ethanol (100 mL) afforded **3d** as a white solid (1.28 g, 91%), m.p. 302–304 °C; ν_max_ (ATR) 818, 1032, 1251, 1556, 1675, 3177, 3381 cm^−1^; δ_H_ (300 MHz, DMSO-*d*_6_) 3.86 (s, 3H), 7.12 (d, *J* = 8.7 Hz, 2H), 8.19 (d, *J* = 3.0 Hz, 1H), 8.26 (d, *J* = 8.7 Hz, 2H), 8.34 (d, *J* = 3.0 Hz, 1H), 12.77 (s, 1H); *m/z* 409 (100, MH^+^); HRMS (ES): MH^+^, found 408.9190. C_15_H_11_N_2_O_2_^79^Br_2_^+^ requires 408.9187.

### 3.3. Oxidative Aromatization of **3a**–**d** with SOCl_2_-DMF Mixture

#### Typical Procedure

*6,8-Dibromo-4-chloro-2-phenylquinazoline* (**4a**). DMF (1 mL) was added dropwise to a stirred suspension of **3a** (1.00 g, 2.60 mmol) in thionyl chloride (30 mL) at room temperature. The mixture was heated under reflux for 2 h and then allowed to cool to room temperature. The mixture was quenched with an ice-cold water and the resulting precipitate was filtered and taken up into chloroform. The chloroform layer was washed with water, dried over MgSO_4_, filtered and evaporated under reduced pressure to afford **4a** as a white solid (0.80 g, 77%), m.p. 169–171 °C; ν_max_ (ATR) 706, 771, 1023, 1297, 1331, 1409, 1456, 1551, 1582 cm^−1^; δ_H_ (300 MHz, CDCl_3_) 7.51–7.56 (m, 3H), 8.30 (d, *J =* 2.1 Hz, 1H), 8.36 (d, *J* = 2.1 Hz, 1H), 8.61–8.65 (m, 2H); δ_C_ (75 MHz, CDCl_3_) 121.2, 123.9, 125.6, 127.6, 128.7, 129.0, 131.8, 135.7, 140.9, 148.1, 160.6, 161.6; *m/z* 397 (100, MH^+^); HRMS (ES): MH^+^, found 396.8733. C_14_H_8_N_2_^35^Cl^79^Br_2_^+^ requires 396.8743.

*6,8-Dibromo-4-chloro-2-(4-fluorophenyl)quinazoline* (**4b**). A stirred suspension of **3b** (1.00 g, 2.48 mmol) and DMF (1 mL) in thionyl chloride (30 mL) was treated as above to afford **4b** as a white solid (0.84 g, 81%), mp. 206–208 °C; ν_max_ (ATR) 721, 1150, 1251, 1300, 1332, 1413, 1508, 1555, 1597 cm^−1^; δ_H_ (300 MHz, CDCl_3_) 7.19 (t, *J* = 8.7 Hz, 2H), 8.30 (d, *J* = 2.1 Hz, 1H), 8.35 (d, *J* = 2.1 Hz, 1H), 8.63 (t, *J* = 8.7 Hz, 2H); δ_C_ (75 MHz, CDCl_3_) 115.8 (d, ^2^*J*_CF_ = 21.7 Hz), 121.3, 123.8, 125.5, 127.6, 131.3 (d, ^3^*J*_CF_ = 8.3 Hz), 132.0 (d, ^4^*J*_CF_ = 3.2 Hz), 141.0, 148.1, 159.6, 161.6, 165.3 (d, ^1^*J*_CF_ = 251.2 Hz); *m/z* 415 (100, MH^+^); HRMS (ES): MH^+^, found 414.8641. C_14_H_7_N_2_F^35^Cl^79^Br_2_^+^ requires 414.8649.

*6,8-Dibromo-4-chloro-2-(4-chlorophenyl)quinazoline* (**4c**). A stirred suspension of **3c** (1.00 g, 2.39 mmol) and DMF (1 mL) in thionyl chloride (30 mL) was treated as above to afford **4c** as a white solid (0.80 g, 77%), m.p. 239–240 °C; ν_max_ (ATR) 744, 786, 68, 1012, 1211, 1298, 1332, 1414, 1553, 1587 cm^−1^; δ_H_ (300 MHz, CDCl_3_) 7.50 (t, *J* = 7.8 Hz, 2H), 8.31 (d, *J* = 2.1 Hz, 1H), 8.36 (d, *J* = 2.1 Hz, 1H), 8.57 (d, *J* = 7.8 Hz, 2H); δ_C_ (75 MHz, CDCl_3_) 121.6, 124.1, 125.6, 127.7, 129.1, 130.3, 134.4, 138.2, 141.1, 148.1, 159.7, 161.8; *m/z* 431 (100, MH^+^); HRMS (ES): MH^+^, found 430.8339. C_14_H_7_N_2_^35^Cl_2_^79^Br_2_^+^ requires 430.8353.

*6,8-Dibromo-4-chloro-2-(4-methoxyphenyl)quinazoline* (**4d**). A stirred suspension of **3d** (1.00 g, 2.41 mmol) and DMF (1 mL) in thionyl chloride (30 mL) was treated as above to afford **4d** as a white solid (1.02 g, 91%), m.p. 200–202 °C; ν_max_ (ATR) 767, 792, 1026, 1164, 1253, 1335, 1422, 1555, 1583 cm^−1^; δ_H_ (300 MHz, CDCl_3_) 3.89 (s, 3H), 7.00 (d, *J* = 8.7 Hz, 2H), 8.25 (d, *J* = 2.1 Hz, 1H), 8.30 (d, *J* = 2.1 Hz, 1H), 8.56 (d, *J* = 8.7 Hz, 2H); δ_C_ (75 MHz, CDCl_3_) 55.4, 114.1, 120.5, 123.6, 125.3, 127.6, 128.5, 130.9, 140.8, 148.2, 160.5, 161.4, 162.8; *m/z* 427 (100, MH^+^); HRMS (ES): MH^+^, found 426.8841. C_15_H_10_N_2_O^35^Cl^79^Br_2_^+^ requires 426.8848.

### 3.4. Sonogashira Cross-Coupling of 4a–d with Terminal Acetylynes

#### Typical Procedure

A mixture of **4** (1 equiv.), Pd(PPh_3_)_4_ (5% of **4**), CuI (5% of **4**) and Cs_2_CO_3_ (1.5 equiv.) in THF (*ca*. 5 mL/mmol of **4**) in a two-necked flask equipped with a stirrer bar, rubber septum and a condenser equipped with a balloon was flushed for 20 min with argon gas. Terminal acetylene (1.2 equiv.) was added to the flask via a syringe and the mixture was flushed for additional 10 min. The mixture was stirred for 24 h at room temperature under argon atmosphere and then quenched with an cold water. The precipitate was filtered on a sintered funnel and then taken-up into chloroform. The solution was dried with MgSO_4_, filtered and then evaporated under reduced pressure. The residue was purified by column chromatography on silica gel to afford the 2-aryl-6,8-dibromo-4-(aryl/alkylethynyl)quinazoline **5**. The following products were prepared in this fashion:

*6,8-Dibromo-2-phenyl-4-(phenylethynyl)quinazoline* (**5a**). A mixture of **4a** (0.50 g, 1.30 mmol), phenylacetylene (0.14 g, 1.40 mmol), Pd(PPh_3_)_4_ (0.07 g, 0.06 mmol), CuI (0.012 g, 0.06 mmol), Cs_2_CO_3_ (0.60 g, 1.70 mmol) in THF (30 mL) afforded **5a** (0.35 g, 60%), m.p. 192–195 °C; *R_f_* (1:1 toluene–petroleum ether) 0.58; ν_max_ (ATR) 684, 730, 777, 870, 1303, 1379, 1525, 2215 cm^−1^; δ_H_ (300 MHz, CDCl_3_) 7.42–7.54 (m, 6H), 7.76 (dd, *J* = 1.2 and 7.5 Hz, 2H), 8.26 (d, *J* = 2.1 Hz, 1H), 8.43 (d, *J* = 2.1 Hz, 1H), 8.67–8.70 (m, 2H); δ_C_ (75 MHz, CDCl_3_) 84.9, 99.1, 120.7, 120.8, 125.4, 125.9, 128.3, 128.6, 128.7, 129.0, 130.5, 131.3, 132.7, 136.9, 140.2. 147.2, 152.3, 161.5; *m/z* 463 (100, MH^+^); HRMS (ES): MH^+^, found 462.9435. C_22_H_13_N_2_^79^Br_2_^+^ requires 462.9445.

*6,8-Dibromo-2-(4-fluorophenyl)-4-(phenylethynyl)quinazoline* (**5b**). A mixture of **4b** (0.50 g, 1.20 mmol), phenylacetylene (0.14 g, 1.40 mmol), Pd(PPh_3_)_4_ (0.07 g, 0.06 mmol), CuI (0.012 g, 0.06 mmol), Cs_2_CO_3_ (0.60 g, 1.70 mmol) in THF (30 mL) afforded **5b** (0.42 g, 72%), m.p. 217–220 °C; *R_f_* (1:1 toluene–petroleum ether) 0.62; ν_max_ (ATR) 688, 721, 760, 801, 842, 866, 1146, 1219, 1308, 1407, 1441, 1491, 1525, 1601, 2210 cm^−1^; δ_H_ (300 MHz, CDCl_3_) 7.20 (t, *J* = 8.7 Hz, 2H), 7.44–7.49 (m, 3H), 7.76 (d, *J* = 7.5 Hz, 2H), 8.29 (d, *J* = 2.1 Hz, 1H), 8.46 (d, *J* = 2.1 1H), 8.70 (t, *J* = 8.7 Hz, 2H); δ_C_ (75 MHz, CDCl_3_) 84.8, 99.3, 115.6 (d, ^2^*J*_CF_ = 21.6 Hz), 120.6, 120.7, 125.3, 125.7, 128.3, 128.5, 1130.6, 131.2 (d, ^3^*J*_CF_ = 8.8 Hz), 132.7, 133.1 (d, ^4^*J*_CF_ = 3.4 Hz), 140.4, 147.2, 152.4, 160.6, 165.1 (d, ^1^*J*_CF_ = 250.2 Hz); *m/z* 481 (100, MH^+^); HRMS (ES): MH^+^, found 480.9347. C_22_H_12_N_2_F^79^Br_2_^+^ requires 480.9351.

*6,8-Dibromo-2-(4-chlorophenyl)-4-(phenylethynyl)quinazoline* (**5c**). A mixture of **4c** (0.50 g, 1.16 mmol), phenylacetylene (0.13 g, 1.29 mmol), Pd(PPh_3_)_4_ (0.07 g, 0.06 mmol), CuI (0.012 g, 0.06 mmol), Cs_2_CO_3_ (0.60 g, 1.70 mmol) in THF (30 mL) afforded **5c** (0.40 g, 69%), m.p. 223–226 °C; *R_f_* (1:1 toluene–petroleum ether) 0.70; ν_max_ (ATR) 682, 735, 748, 801, 870, 1089, 1308, 1375, 1408, 1525, 1544, 2210 cm^−1^; δ_H_ (300 MHz, CDCl_3_) 7.45–7.51 (m, 5H), 7.77 (d, *J* = 6.0 Hz, 2H), 8.29 (d, *J* = 1.8 Hz, 1H), 8.47 (d, *J* = 1.8 Hz, 1H), 8.64 (d, *J* = 8.7 Hz, 2H); δ_C_ (75 MHz, CDCl_3_) 84.8, 99.4, 120.7, 121.0, 125.5, 125.8, 128.3, 128.8, 128.9, 130.3, 130.6, 132.7, 135.4, 137.7, 140.4, 147.2, 152.4, 160.6; *m/z* 497 (100, MH^+^); HRMS (ES): MH^+^, found 496.8040. C_22_H_12_N_2_^35^Cl^79^Br_2_^+^ requires 496.8056.

*6,8-Dibromo-2-(4-methoxyphenyl)-4-(phenylethynyl)quinazoline* (**5d**). A mixture of **4d** (0.50 g, 1.20 mmol), phenylacetylene (0.12 g, 1.20 mmol), Pd(PPh_3_)_4_ (0.07 g, 0.06 mmol), CuI (0.012 g, 0.06 mmol), Cs_2_CO_3_ (0.60 g, 1.70 mmol) in THF (30 mL) afforded **5d** (0.42 g, 72%), m.p. 195–198 °C; *R_f_* (1:1 toluene–petroleum ether) 0.30; ν_max_ (ATR) 684, 754, 801, 1024, 1162, 1256, 1309, 1411, 1543, 1608, 2210 cm^−1^; δ_H_ (300 MHz, CDCl_3_) 3.90 (s, 3H), 7.03 (d, *J* = 7.8 Hz, 2H), 7.39–7.49 (m, 3H), 7.76 (d, *J* = 7.0 Hz, 2H), 8.24 (s, 1H), 8.42 (s, 1H), 8.64 (d, *J* = 7.8 Hz, 2H); δ_C_ (75 MHz, CDCl_3_) 55.4, 85.0, 98.7, 114.0, 120.0, 120.8, 125.1, 125.6, 128.3, 128.7, 129.9, 130.5, 130.8, 132.7, 140.1, 147.4, 152.2, 161.4, 162.4; *m/z* 493 (100, MH^+^); HRMS (ES): MH^+^, found 492.9541. C_23_H_15_N_2_O^79^Br_2_^+^ requires 492.9551.

*6,8-Dibromo-2-phenyl-4-(pyridin-2-ethynyl)quinazoline* (**5e**). A mixture of **4a** (0.50 g, 1.30 mmol), 2-ethynylpyridine (0.14 g, 1.30 mmol), Pd(PPh_3_)_4_ (0.07 g, 0.06 mmol), CuI (0.012 g, 0.06 mmol), Cs_2_CO_3_ (0.60 g, 1.70 mmol) in THF (30 mL) afforded **5e** (0.36 g, 65%), m.p. 207–209 °C; *R_f_* (toluene) 0.15; ν_max_ (ATR) 703, 734, 782, 892, 1304, 1464, 1544, 2227 cm^−1^; δ_H_ (300 MHz, CDCl_3_) 7.54–7.55 (m, 4H), 7.77–7.85 (m, 2H), 8.30 (d, *J* = 1.8 Hz, 1H), 8.54 (d, *J* = 1.8 Hz, 1H), 8.68–8.82 (m, 2H), 8.76 (d, *J =* 4.8 Hz, 1H); δ_C_ (75 MHz, CDCl_3_) 83.5, 96.4, 121.1, 124.5, 125.5, 125.9, 128.2, 128.6, 128.7, 130.0, 131.4, 136.5, 136.8, 140.6, 141.5, 147.4, 150.6, 151.7, 161.5; *m/z* 464 (100, MH^+^); HRMS (ES): MH^+^, found 463.9398. C_21_H_12_N_3_^79^Br_2_^+^ requires 463.9398.

*6,8-Dibromo-2-(4-fluorophenyl)-4-(pyridin-2-ethynyl)quinazoline* (**5f**). A mixture of **4b** (0.50 g, 1.16 mmol), 2-ethynylpyridine (0.14 g, 1.30 mmol), Pd(PPh_3_)_4_ (0.07 g, 0.06 mmol), CuI (0.012 g, 0.06 mmol), Cs_2_CO_3_ (0.60 g, 1.70 mmol) in THF (30 mL) afforded **5f** (0.38 g, 67%), m.p. 216–218 °C; *R_f_* (toluene) 0.20; ν_max_ (ATR) 702, 777, 848, 1150, 1210, 1307, 1374, 1409, 1542, 2220 cm^−1^; δ_H_ (300 MHz, CDCl_3_) 7.20 (d, *J* = 8.7 Hz, 2H), 7.39–7.44 (m, 1H), 7.77–7.85 (m, 2H), 8.29 (d, *J* = 1.8 Hz, 1H), 8.52 (d, *J* = 1.8 Hz, 1H), 8.70 (d, *J* = 8.7 Hz, 2H), 8.75 (d, *J* = 4.5 Hz, 1H); δ_C_ (75 MHz, CDCl_3_) 83.4, 96.6, 115.6 (d, ^2^*J*_CF_ = 21.6 Hz), 121.1, 124.6, 125.4, 125.7, 128.3, 128.6, 131.3 (d, ^2^*J*_CF_ = 8.8 Hz), 133.1 (d, ^3^*J*_CF_ = 3.4 Hz), 136.5, 140.7, 141.4, 147.4, 150.7, 151.7, 160.6, 165.1 (d, ^1^*J*_CF_ = 250.2 Hz); *m/z* 482 (100, MH^+^); HRMS (ES): MH^+^, found 481.9301. C_21_H_11_N_3_F^79^Br_2_^+^ requires 481.9304.

*6,8-Dibromo-2-(4-methoxyphenyl)-4-(pyridin-2-ethynyl)quinazoline* (**5g**). A mixture of **4d** (0.50 g, 1.18 mmol), 2-ethynylpyridine (0.14 g, 1.30 mmol), Pd(PPh_3_)_4_ (0.07 g, 0.06 mmol), CuI (0.012 g, 0.06 mmol), Cs_2_CO_3_ (0.60 g, 1.70 mmol) in THF (30 mL) afforded **5g** (0.32 g, 55%), m.p. 204–206 °C; *R_f_* (toluene) 0.13; ν_max_ (ATR) 802, 1023, 1163, 1258, 1309, 1412, 1525, 1582, 1608, 2221 cm^−1^; δ_H_ (300 MHz, CDCl_3_) 3.90 (s, 3H), 7.03 (d, *J* = 8.7 Hz, 2H), 7.39–7.43 (m, 1H), 7.77–7.79 (m, 2H), 8.27 (d, *J* = 1.8 Hz, 1H), 8.50 (d, *J* = 1.8 Hz, 1H), 8.65 (d, *J* = 8.7 Hz, 2H), 8.75 (d, *J* = 4.5 Hz, 1H); δ_C_ (75 MHz, CDCl_3_) 55.4, 83.6, 96.1, 114.0, 120.3, 124.5, 125.1, 125.6, 128.2, 128.5, 129.5, 130.8, 136.4, 140.4, 141.5, 147.4, 150.7, 151.4, 161.3, 162.4; *m/z* 494 (100, MH^+^); HRMS (ES): MH^+^, found 493.9517. C_22_H_14_N_3_O^79^Br_2_^+^ requires 493.9504.

*6,8-Dibromo-4-(3-hydroxybutynyl)-2-phenylquinazoline* (**5h**). A mixture of **4a** (0.42 g, 1.10mmol), 3-butyn-2-ol (0.09 g, 1.32 mmol), Pd(PPh_3_)_4_ (0.07 g, 0.06 mmol), CuI (0.012 g, 0.06 mmol), Cs_2_CO_3_ (0.60 g, 1.70 mmol) in THF (30 mL) afforded **5h** (0.28 g, 56%), m.p. 162–165 °C; *R_f_* (1:1, ethyl acetate–hexane) 0.70; ν_max_ (ATR) 683, 703, 735, 777, 868, 1025, 1073, 1304, 1364, 1458, 1529, 2222, 3373 cm^−1^; δ_H_ (300 MHz, CDCl_3_) 1.68 (dq, *J* = 5.1 and 6.6 Hz, 3H), 2.97 (d, *J* = 5.1 Hz, 1H), 4.91 (q, *J* = 6.6 Hz, 1H), 7.50–7.53 (m 3H), 8.26 (d, *J* = 1.8 Hz, 1H), 8.30 (d, *J* = 1.8 Hz, 1H), 8.62–8.66 (m, 2H); δ_C_ (75 MHz, CDCl_3_) 23.6, 58.7, 79.4, 100.9, 120.8, 125.0, 125.7, 128.0, 128.6, 128.9, 131.4, 136.6, 140.3, 147.0, 151.6, 161.3; *m/z* 433 (100, MH^+^); HRMS (ES): MH^+^, found 432.9371. C_18_H_14_N_3_O^79^Br_2_^+^ requires 432.9371.

### 3.5. Typical Procedure for the Suzuki-Miyaura Cross-Coupling of **5a**–**d** with Arylboronic Acids

*2,6,8-Triphenyl-4-(phenylethynyl)quinazoline* (**6a**). A mixture of **5a** (0.30 g, 0.64 mmol), PdCl_2_(PPh_3_)_2_ (0.022 g, 0.03 mmol), PCy_3_ (0.02 g, 0.06 mmol) and K_2_CO_3_ (0.23 g, 1.60 mmol) in dioxane-water (3:1, v/v; 20 mL) in a three-necked flask equipped with a stirrer, condenser and a rubber septum was flushed with nitrogen gas for 20 min. Phenylboronic acid (0.19 g, 1.50 mmol) was added to the flask via a syringe. The mixture was flushed for additional 10 min and a balloon filled with argon gas was connected to the top of the condenser. The mixture was heated with stirring at 100 °C for 5 h under nitrogen atmosphere and then allowed to cool to room temperature. The cooled mixture was added to a beaker containing an ice-cold water and the product was extracted into ethyl acetate. The combined organic layers were dried over anhydrous MgSO_4_, filtered, and evaporated under reduced pressure. The residue was purified by column chromatography on silica gel to afford **6a** as a solid (0.179 g, 61%), m.p. 184–186 °C; *R_f_* (1:1 toluene–hexane) 0.41; ν_max_ (ATR) 688, 756, 1396, 1491, 1535, 1562, 2208 cm^−1^; δ_H_ (300 MHz, CDCl_3_) 7.46–7.61 (m, 12H), 7.77–7.82 (m, 4H), 7.90 (d, *J =* 7.5 Hz, 2H), 8.25 (d. *J* = 2.1 Hz, 1H), 8.57–8.60 (m, 3H); δ_C_ (75 MHz, CDCl_3_) 86.0, 97.8, 121.4, 123.3, 124.6, 127.5, 127.8, 128.0, 128.2, 128.5, 128.6, 128.7, 129.2, 130.1, 130.6, 131.0, 132.7, 134.3, 137.8, 137.9, 139.9, 140.1, 140.7, 148.0, 153.1, 160.0; *m/z* 459 (100, MH^+^); HRMS (ES): MH^+^, found 459.1870. C_34_H_23_N_2_^+^ requires 459.1861.

*2-(4-Fluorophenyl)-6,8-diphenyl-4-(phenylethynyl)quinazoline* (**6b**). A mixture of **5b** (0.20 g, 0.43 mmol), phenylboronic acid (0.10 g, 1.60 mmol), PdCl_2_(PPh_3_)_2_ (0.022 g, 0.03 mmol), PCy_3_ (0.02 g, 0.06 mmol) and K_2_CO_3_ (0.23 g, 1.60 mmol) in dioxane-water (20 mL) afforded **6b** (0.12 g, 57%), m.p. 250–252 °C; *R_f_* (1:1 toluene–hexane) 0.54; ν_max_ (ATR) 686, 741, 846, 1148, 1218, 1409, 1536, 1536, 1598, 2210 cm^−1^; δ_H_ (300 MHz, CDCl_3_) 7.14 (d, *J* = 8.7 Hz, 2H), 7.42–7.60 (m, 9H), 7.75–7.81 (m, 4H), 7.87 (d, *J* = 6.9 Hz, 2H), 8.22 (d, *J* = 1.8 Hz, 1H), 8.55 (t, *J* = 8.7 Hz, 2H), 8.56 (d, *J* = 1.8 Hz, 1H); δ_C_ (75 MHz, CDCl_3_) 85.9, 98.0, 115.3 (d, ^2^*J*_CF_ = 21.6 Hz), 121.3, 123.3, 124.4, 127.5, 127.8, 128.0, 128.2, 128.7, 129.2, 130.2, 130.8 (d, ^3^*J*_CF_ = 8.9 Hz), 130.9, 132.6, 134.0 (d, ^4^*J*_CF_ = 3.3 Hz), 134.4, 137.9, 139.8, 140.1, 140.7, 147.9, 153.1, 159.1, 164.5 (d, ^1^*J*_CF_ = 248.8 Hz); *m/z* 477 (100, MH^+^); HRMS (ES): MH^+^, found 477.1772. C_34_H_22_N_2_F^+^ requires 477.1767.

*2-(4-Chlorophenyl)-6,8-diphenyl-4-(2-phenylethynyl)quinazoline* (**6c**). A mixture of **5c** (0.20 g, 0.41 mmol), phenylboronic acid (0.12 g, 1.00 mmol), PdCl_2_(PPh_3_)_2_ (0.022 g, 0.03 mmol), PCy_3_ (0.02 g, 0.06 mmol) and K_2_CO_3_ (0.23 g, 1.60 mmol) in dioxane-water (20 mL) afforded **6c** (0.18 g, 88%), m.p. 246–248 °C; *R_f_* (1:1 toluene–hexane) 0.60; ν_max_ (ATR) 687, 739, 752, 1012, 1087, 1533, 1576, 2211 cm^−1^; δ_H_ (300 MHz, CDCl_3_) 7.41–7.60 (m, 11H), 7.75–7.81 (m, 4H), 7.90 (d, *J* = 6.9 Hz, 2H), 8.22 (d, *J* = 1.8 Hz, 1H), 8.50 (d, *J* = 8.4 Hz, 2H), 8.55 (d, *J* = 1.8 Hz, 1H); δ_C_ (75 MHz, CDCl_3_) 85.9, 98.0, 121.3, 123.3, 124.5, 127.5, 127.9, 128.0, 128.3, 128.6. 128.7, 129.2, 130.0, 130.2, 130.9, 132.6, 134.4, 136.3, 136.7, 137.8, 139.8, 140.3, 140.7, 147.8, 153.1, 159.0; *m/z* 493 (100, MH^+^); HRMS (ES): MH^+^, found 493.1475. C_34_H_22_N_2_^35^Cl^+^ requires 493.1472.

*2-(4-Methoxyphenyl)-6,8-diphenyl-4-(phenylethynyl)quinazoline* (**6d**). A mixture of **5d** (0.20 g, 0.42 mmol), phenylboronic acid (0.100 g, 1.20 mmol), PdCl_2_(PPh_3_)_2_ (0.015 g, 0.02 mmol), PCy_3_ (0.013 g, 0.04 mmol) and K_2_CO_3_ (0.15 g, 1.30 mmol) in dioxane-water (20 mL) afforded **6d** (0.14 g, 68%), m.p. 220–223 °C; *R_f_* (1:1 toluene–hexane) 0.17; ν_max_ (ATR) 685, 699, 752, 1028, 1161, 1247, 1411, 1533, 1602, 2205 cm^−1^; δ_H_ (300 MHz, CDCl_3_) 3.88 (s, 3H), 6.99 (d, *J* = 8.7 Hz, 2H), 7.44–7.60 (m, 9H), 7.76–7.81 (m, 4H), 7.90 (d, *J* = 6.9 Hz, 2H), 8.21 (d, *J* = 2.1 Hz, 1H), 8.53 (d, *J* = 8.7 Hz, 2H), 8.55 (d, *J* = 2.1 Hz, 1H); δ_C_ (75 MHz, CDCl_3_) 55.3, 86.0, 97.5, 113.8, 121.5, 123.4, 124.3, 127.4, 127.7, 127.9, 128.1, 128.6, 129.2, 130.0, 130.4, 130.6, 131.0, 132.6, 134.2, 138.0, 139.6, 140.0, 140.5, 148.0, 153.0, 159.9, 161.8; *m/z* 489 (100, MH^+^); HRMS (ES): MH^+^, found 489.1976. C_35_H_25_N_2_O^+^ requires 489.1967.

*6,8-Bis(4-fluorophenyl)-2-phenyl-4-(phenylethynyl)quinazoline* (**6e**). A mixture of **5a** (0.20 g, 0.43 mmol), 4-fluorophenylboronic acid (0.15 g, 1.07 mmol), PdCl_2_(PPh_3_)_2_ (0.015 g, 0.02 mmol), PCy_3_ (0.01 g, 0.04 mmol) and K_2_CO_3_ (0.12 g, 1.30 mmol) in dioxane-water (20 mL) afforded **6e** (0.14 g, 64%), m.p. 228–230 °C; *R_f_* (1:1 toluene–hexane) 0.48; ν_max_ (ATR) 691, 725, 825, 1158, 1232, 1466, 1510, 1561, 1605, 2206 cm^−1^; δ_H_ (300 MHz, CDCl_3_) 7.23 (t, *J* = 8.7 Hz, 2H), 7.26 (t, *J* = 8.7 Hz, 2H), 7.46–7.50 (m, 6H), 7.72–7.80 (m, 4H), 7.85 (t, *J* = 8.7 Hz, 2H), 8.12 (d, *J =* 2.1 Hz, 1H), 8.50 (d, *J* = 2.1 Hz, 1H), 8.56 (t, *J* = 8.7 Hz, 2H); δ_C_ (75 MHz, CDCl_3_) 85.9, 98.0, 115.0 (d, ^2^*J*_CF_ = 21.4 Hz), 116.2 (d, ^2^*J*_CF_ = 21.4 Hz), 121.3, 123.2, 124.5, 128.6 (d, ^3^*J*_CF_ = 8.3 Hz), 128.7, 129.1 (d, ^3^*J*_CF_ = 8.3 Hz), 130.2, 130.7, 132.5, 132.6, 133.7 (d, ^4^*J*_CF_ = 3.2 Hz), 133.8, 135.9 (d, ^4^*J*_CF_ = 3.2 Hz), 137.7, 139.1, 139.8, 147.8, 153.1, 160.1, 162.7 (d, ^1^*J*_CF_ = 245.9 Hz), 163.0 (d, ^1^*J*_CF_ = 247.0 Hz); *m/z* 495 (100, MH^+^); HRMS (ES): MH^+^, found 495.1685. C_34_H_21_N_2_F_2_^+^ requires 495.1673.

*2,6,8-Tris(4-fluorophenyl)-4-(phenylethynyl)quinazoline* (**6f**). A mixture of **5b** (0.20 g, 0.41 mmol), 4-fluorophenylboronic acid (0.15 g, 1.07 mmol), PdCl_2_(PPh_3_)_2_ (0.015 g, 0.021 mmol), PCy_3_ (0.012 g, 0.04 mmol) and K_2_CO_3_ (0.15 g, 1.60 mmol) in dioxane-water (20 mL) afforded **6f** (0.16 g, 76%), m.p. 255–257 °C; *R_f_* (1:1 toluene–hexane) 0.58; ν_max_ (ATR) 686, 751, 809, 824, 1147, 1232, 1411, 1462, 1492, 1561, 1599, 2208 cm^−1^; δ_H_ (300 MHz, CDCl_3_) 7.15 (t, *J* = 8.7 Hz, 2H), 7.23 (t, *J* = 8.7 Hz, 2H), 7.26 (t, *J* = 8.7 Hz, 2H), 7.43–7.51 (m, 3H), 7.74 (t, *J* = 8.7 Hz, 2H), 7.75–7.78 (m, 2H), 7.82 (t, *J* = 8.7 Hz, 2H), 8.11 (d, *J =* 2.1 Hz, 1H), 8.48 (d, *J* = 2.1 Hz, 1H), 8.54 (t, *J* = 8.7 Hz, 2H); δ_C_(75 MHz, CDCl_3_) 85.8, 98.1, 115.0 (d, ^2^*J*_CF_ = 21.3 Hz), 115.5 (d, ^2^*J*_CF_ = 21.7 Hz), 116.2 (d, ^2^*J*_CF_ = 21.6 Hz), 121.2, 123.2, 124.4, 128.7, 129.1 (d, ^3^*J*_CF_ = 8.3 Hz), 130.3, 130.7 (d, ^3^*J*_CF_ = 8.8 Hz), 132.4 (d, ^3^*J*_CF_ = 8.0 Hz), 132.5, 133.6 (d, ^4^*J*_CF_ = 3.2 Hz), 133.8 (d, ^4^*J*_CF_ = 3.0 Hz), 133.9, 135.8 (d, ^4^*J*_CF_ = 3.2 Hz), 139.1, 139.8, 147.7, 153.2, 159.2, 162.7 (d, ^1^*J*_CF_ = 245.9 Hz), 163.0 (d, ^1^*J*_CF_ = 247.0 Hz), 164.7 (d, ^1^*J*_CF_ = 249.0 Hz); *m/z* 513 (100, MH^+^); HRMS (ES): MH^+^, found 513.1585. C_34_H_20_N_2_F_3_^+^ requires 513.1579.

*2-(4-Chlorophenyl)-6,8-bis(4-fluorophenyl)-4-(phenylethynyl)quinazoline* (**6g**). A mixture of **5c** (0.20 g, 0.42 mmol), 4-fluorophenylboronic acid (0.15 g, 1.07 mmol), PdCl_2_(PPh_3_)_2_ (0.015 g, 0.02 mmol), PCy_3_ (0.01 g, 0.04 mmol) and K_2_CO_3_ (0.12 g, 1.30 mmol) in dioxane-water (20 mL) afforded **6g** (0.17 g, 77%), m.p. 243–245 °C; *R_f_* (1:1 toluene–hexane) 0.72; ν_max_ (ATR) 747, 809, 820, 1011, 1088, 1158, 1231, 1509, 1605, 2205 cm^−1^; δ_H_ (300 MHz, CDCl_3_) 7.24 (t, *J* = 8.7 Hz, 2H), 7.26 (t, *J* = 8.7 Hz, 2H), 7.43–7.51 (m, 3H), 7.74 (t, *J* = 8.7 Hz, 2H), 7.75–7.80 (m, 2H), 7.82 (t, *J* = 8.7 Hz, 2H), 8.12 (d, *J* = 2.4 Hz, 1H), 8.48 (d, *J* = 2.4 Hz, 1H), 8.48–8.49 (m, 2H); δ_C_ (75 MHz, CDCl_3_) 85.7, 98.2, 114.9 (d, ^2^*J*_CF_ = 21.3 Hz), 116.2 (d, ^2^*J*_CF_ = 21.4 Hz), 121.2, 123.1, 124.4, 128.7, 128.8, 129.1 (d, ^3^*J*_CF_ = 8.3 Hz), 129.9, 130.3, 132.4 (d, ^3^*J*_CF_ = 8.3 Hz), 132.5, 133.6 (d, ^4^*J*_CF_ = 3.4 Hz), 133.9, 135.8 (d, ^4^*J*_CF_ = 3.4 Hz), 136.1, 136.9, 139.2, 139.7, 147.6, 153.1, 159.1, 162.8 (d, ^1^*J*_CF_ = 246.2 Hz), 163.0 (d, ^1^*J*_CF_ = 247.0 Hz); *m/z* 529 (100, MH^+^); HRMS (ES): MH^+^, found 529.1282. C_34_H_20_N_2_F_2_^35^Cl^+^ requires 529.1283.

*6,8-Bis(4-fluorophenyl)-2-(4-methoxyphenyl)-4-(phenylethynyl)quinazoline* (**6h**). A mixture of **5d** (0.20 g, 0.42 mmol), 4-fluorophenylboronic acid (0.15 g, 1.07 mmol), PdCl_2_(PPh_3_)_2_ (0.015 g, 0.02 mmol), PCy_3_ (0.01 g, 0.04 mmol) and K_2_CO_3_ (0.12 g, 1.30 mmol) in dioxane-water (20 mL) afforded **6h** (0.18 g, 58%), m.p. 211–230 °C; *R_f_* (1:1 toluene–hexane) 0.50; ν_max_ (ATR) 684, 750, 810, 1028, 1157, 1227, 1252, 1507, 1537, 1605, 2203 cm^−1^; δ_H_ (300 MHz, CDCl_3_) 3.88 (s, 3H), 7.00 (d, *J* = 8.7 Hz, 2H), 7.22 (d, *J* = 8.7 Hz, 2H), 7.25 (d, *J* = 8.7 Hz, 2H), 7.44–7.50 (m, 2H), 7.75–7.84 (m, 5H), 8.09 (d, *J* = 2.1 Hz, 1H); 8.48 (d, *J* = 2.1 Hz, 1H), 8.59 (dd, *J* = 2.4 and 7.5 Hz, 2H); δ_C_ (75 MHz, CDCl_3_) 55.4, 85.9, 97.7, 113.9, 114.9 (d, ^2^*J*_CF_ = 21.4 Hz), 116.1 (d, ^2^*J*_CF_ = 21.4 Hz), 121.4, 123.2, 124.2, 128.7, 129.1 (d, ^3^*J*_CF_ = 8.3 Hz), 130.1, 130.3, 131.8, 132.5 132.6 (d, ^3^*J*_CF_ = 8.4 Hz), 133.7, 133.8 (d, ^4^*J*_CF_ = 3.0 Hz), 136.1 (d, ^4^*J*_CF_ = 3.0 Hz), 138.5, 139.5, 147.9, 153.0, 160.0, 161.9, 162.8 (d, ^1^*J*_CF_ = 245.9 Hz), 162.9 (d, ^1^*J*_CF_ = 246.5 Hz); *m/z* 525 (100, MH^+^); HRMS (ES): MH^+^, found 525.1785. C_35_H_23_N_2_OF_2_^+^ requires 525.1778.

*6,8-Bis(4-methoxyphenyl)-2-phenyl-4-(phenylethynyl)quinazoline* (**6i**). A mixture of **5a** (0.30 g, 0.64 mmol), 4-methoxyphenylboronic acid (0.20 g, 1.60 mmol), PdCl_2_(PPh_3_)_2_ (0.022 g, 0.03 mmol), PCy_3_ (0.02 g, 0.06 mmol) and K_2_CO_3_ (0.23 g, 1.60 mmol) in dioxane-water (20 mL) afforded **6i** (0.23 g, 69%), m.p. 209–211 °C; *R_f_* (2:1 toluene–hexane) 0.28; ν_max_ (ATR) 691; 831, 1032, 1174, 1243, 1393, 1460, 1510, 1608, 2209 cm^−1^; δ_H_ (300 MHz, CDCl_3_) 3.88 (s, 3H), 3.93 (s, 3H), 7.07 (d, *J* = 8.7 Hz, 2H), 7.11 (d, *J* = 8.7 Hz, 2H), 7.44–7.50 (m, 6H), 7.73 (d, *J* = 8.7 Hz, 2H), 7.77–7.81 (m, 2H), 7.86 (d, *J* = 8.7 Hz, 2H), 8.17 (d, *J* = 2.1 Hz, 1H); 8.48 (d, *J* = 2.1 Hz, 1H), 8.59 (dd, *J* = 2.4 and 7.5 Hz, 2H); δ_C_ (75 MHz, CDCl_3_) 55.3 (2×C), 86.1, 97.5, 113.4, 114.5, 121.4, 121.7, 124.6, 126.4 (2×C), 128.6 (2×C), 130.0, 130.3, 130.4, 132.1, 132.2, 132.5, 133.4, 137.8, 139.5, 140.0, 147.6, 152.7, 159.4, 159.5, 159.7; *m/z* 519 (100, MH^+^); HRMS (ES): MH^+^, found 519.2071. C_36_H_27_N_2_O_2_^+^ requires 519.2073.

*2-(4-Fluorophenyl)-6,8-bis(4-methoxyphenyl)-4-(phenylethynyl)quinazoline* (**6j**). A mixture of **5b** (0.40 g, 0.86 mmol), 4-methoxyphenylboronic acid (0.26 g, 2.15 mmol), PdCl_2_(PPh_3_)_2_ (0.03 g, 0.04 mmol), PCy_3_ (0.024 g, 0.08 mmol) and K_2_CO_3_ (0.30 g, 2.15 mmol) in dioxane-water (30 mL) afforded **6j** (0.29 g, 62%), m.p. 236–238 °C; *R_f_* (2:1 toluene–hexane) 0.33; ν_max_ (ATR) 831, 1017, 1150, 1222, 1287, 1508, 1605, 2208 cm^−1^; δ_H_ (300 MHz, CDCl_3_) 3.89 (s, 3H), 3.94 (s, 3H), 7.07 (d, *J* = 8.7 Hz, 2H), 7.11 (d, *J* = 8.7 Hz, 2H), 7.15 (d, *J* = 8.7 Hz, 2H), 7.44–7.49 (m, 3H), 7.73 (d, *J* = 8.7 Hz, 2H), 7.75–7.80 (m, 2H), 7.83 (d, *J* = 8.7 Hz, 2H), 8.14 (d, *J* = 2.4 Hz, 1H), 8.46 (d, *J* = 1.8 Hz, 1H), 8.57 (t, *J* = 8.7 Hz, 2H); δ_C_ (75 MHz, CDCl_3_) 55.3, 55.4, 86.0, 97.8, 113.5, 114.6, 115.3 (d, ^2^*J*_CF_ = 21.3 Hz), 121.4, 124.6, 128.5, 128,7, 130.1, 130.3, 130.7 (d, ^3^*J*_CF_ = 8.5 Hz), 132.1, 132.3, 132.5, 133.7, 134.1 (d, ^4^*J*_CF_ = 3.2 Hz), 139.7, 140.1, 147.6, 152.8, 158.7, 159.4, 159.8, 164.6 (d, ^1^*J*_CF_ = 248.7 Hz); m/z 537 (100, MH^+^); HRMS (ES): MH^+^, found 537.1986. C_36_H_26_N_2_FO_2_^+^ requires 537.1978.

*2-(4-Chlorophenyl)-6,8-bis(4-methoxyphenyl)-4-(phenylethynyl)quinazoline* (**6k**). A mixture of **5c** (0.30 g, 0.64 mmol), 4-methoxyphenylboronic acid (0.20 g, 1.60 mmol), PdCl_2_(PPh_3_)_2_ (0.023 g, 0.03 mmol), PCy_3_ (0.020 g, 0.06 mmol) and K_2_CO_3_ (0.23 g, 1.60 mmol) in dioxane-water (20 mL) afforded **6k** (0.27 g, 76%), m.p. 233–235 °C; *R_f_* (2:1 toluene–hexane) 0.38; ν_max_ (ATR) 746, 807, 830, 1016, 1178, 1248, 1491, 1511, 1605, 2210 cm^−1^; δ_H_ (300 MHz, CDCl_3_) 3.88 (s, 3H), 3.92 (s, 3H), 7.05 (d, *J* = 8.7 Hz, 2H), 7.10 (d, *J* = 8.7 Hz, 2H), 7.41–7.47 (m, 5H), 7.71 (d, *J* = 8.7 Hz, 2H), 7.76 (dd, *J* = 1.8 and 7.8 Hz, 2H), 7.81 (d, *J* = 8.7 Hz, 2H), 8.15 (d, *J* = 1.8 Hz, 1H); 8.44 (d, *J* = 1.8 Hz, 1H), 8.49 (d, *J* = 8.4 Hz, 2H); δ_C_ (75 MHz, CDCl_3_) 55.3, 55.4, 86.0, 97.9, 113.5, 114.6, 121.4, 121.9, 124.7, 128.5, 128.6, 128.7, 129.9, 130.1, 130.3, 132.1, 132.3, 132.1, 132.3, 132.5, 133.7, 136.4, 136.6, 139.9, 140.2, 147.6, 152.9, 158.6, 159.5, 159.9; *m/z* 553 (100, MH^+^); HRMS (ES): MH^+^, found 553.1689. C_36_H_26_N_2_O_2_^35^Cl^+^ requires 553.1683.

*2,6,8-Tris(4-methoxyphenyl)-4-(phenylethynyl)quinazoline* (**6l**). A mixture of **5d** (0.30 g, 0.64 mmol), 4-methoxyphenylboronic acid (0.20 g, 1.60 mmol), PdCl_2_(PPh_3_)_2_ (0.02 g, 0.03 mmol), PCy_3_ (0.02 g, 0.06 mmol) and K_2_CO_3_ (0.24 g, 1.61 mmol) in dioxane-water (20 mL) afforded **6l** (0.18 g, 52%), m.p. 237–239 °C; *R_f_* (2:1 toluene–hexane) 0.20; ν_max_ (ATR) 753, 808, 832, 1018, 1162, 1176, 1243, 1507, 1605, 2207 cm^−1^; δ_H_ (300 MHz, CDCl_3_) 3.87 (s, 3H), 3.88 (s, 3H), 3.93 (s, 3H), 6.99 (d, *J* = 8.7 Hz, 2H), 7.04 (d, *J* = 8.7 Hz, 2H), 7.10 (d, *J* = 8.7 Hz, 2H), 7.74–7.47 (m, 3H), 7.71 (d, *J* = 8.7 Hz, 2H), 7.75–7.78 (m, 2H), 7.84 (d, *J* = 8.7 Hz, 2H), 8.12 (d, *J* = 1.8 Hz, 1H); 8.42 (d, *J* = 1.8 Hz, 1H), 8.52 (d, *J* = 8.7 Hz, 2H);δ_C_ (75 MHz, CDCl_3_) 55.3, 55.4, 55.5, 86.1, 97.3, 113.4, 113.7, 114.5, 121.5, 121.9, 124.3, 128.4, 128.6, 129.9, 130.2, 130.4, 130.6, 132.1, 132.4, 132.5, 133.4, 139.1, 139.8, 147.7, 152.7, 159.3, 159.4, 159.7, 161.6; *m/z* 549 (100, MH^+^); HRMS (ES): MH^+^, found 549.2192. C_37_H_29_N_2_O_3_^+^ requires 549.2178.

## 4. Conclusions

Elaboration of the 6,8-dibromo-4-chloroquinazoline scaffold via sequential Sonogashira and Suzuki-Miyaura cross-coupling reactions with terminal alkynes and arylboronic acids afforded novel polysubstituted quinazoline derivatives that would not be readily accessible otherwise. Exclusive replacement of 4-chloro atom of the 6,8-dibromo-4-chloroquinazolines via Sonogashira cross-coupling with stoichiometric amount of terminal alkynes is attributed to the α-nitrogen effect, which makes the 4-position highly activated than other positions. Lack of selectivity during Suzuki cross-coupling of the 2-aryl-4-alkynyl-6,8-dibromoquinazolines with arylboronic acids, on the other hand, is presumably the consequence of comparable C(6)–Br and C(8)–Br bond dissociation energies. The polyaryl substituted heterocycles **6** comprise an electron-deficient quinazoline framework as an electron-acceptor linked to the aryl rings directly or through a π-conjugated bridge to comprise donor-π-acceptor systems. Preliminary photophysical (absorption and emission) properties of these compounds showed a strong correlation with the substituents on the 2-, 6- and 8-phenyl groups. Based on the orbital diagrams, the electronic transitions of compounds **6** can be attributed to ICT from the aryl substituents to the quinazoline ring. Due to its electron deficiency, the quinazoline moiety may provide a site for reduction in this D-π-A system. This makes quinazolines **6a**–**l** suitable candidates for further studies using cyclic voltametry to probe oxidation and reduction potentials and the stability of the oxidized and reduced forms. Compounds **6a**–**l**, on the other hand, can be used as substrates for the synthesis of metal complexes with iridium, palladium or platinum, for example, as a prelude to compounds with potential application as organic light-emitting diode in materials. Moreover, the analogous 2-substituted quinazolines bearing alkynyl substituent on the C-4 or C-6 position exhibit excellent EGFR or Aurora A kinase inhibition activity [23].
